# Is a Femoro-Acetabular Impingement Type Cam Predictable after Slipped Capital Femoral Epiphysis?

**DOI:** 10.3390/children8110992

**Published:** 2021-11-02

**Authors:** Nils Wirries, Gesche Heinrich, Alexander Derksen, Stefan Budde, Thilo Floerkemeier, Henning Windhagen

**Affiliations:** 1 Department of Orthopaedic Surgery at Diakovere Annastift, Hannover Medical School, 30625 Hannover, Germany; Nils.Wirries@diakovere.de (N.W.); Gesche.M.Heinrich@stud.mh-hannover.de (G.H.); Alexander.Derksen@diakovere.de (A.D.); st-budde@t-online.de (S.B.); 2 go:h Gelenkchirurgie-Orthopädie: Hannover, 30159 Hannover, Germany; thilo.floerkemeier@g-o-hannover.de

**Keywords:** slipped capital femoral epiphysis, femoro-acetabular impingement, prognostic factor, correlation

## Abstract

(1) Background: Previous studies have proven a high incidence of a femoro-acetabular impingement (FAI) type cam in patients sustaining a slipped capital femoral epiphysis (SCFE). Thus, the current study analyzed, if a cam deformity is predictable after SCFE treatment; (2) Methods: 113 cases of SCFE were treated between 1 January 2005 and 31 December 2017. The radiological assessment included the slip angle after surgery (referenced to the femoral neck (epiphyseal tilt) and shaft axis as Southwick angle) and the last available lateral center edge angle (LCEA), the acetabular- and alpha angle. A correlation was performed between these parameters and the last alpha angle to predict a FAI type cam; (3) Results: After a mean follow-up of 4.3 years (±1.9; 2.0–11.2), 48.5% of the patients showed a FAI type cam and 43.2% a dysplasia on the affected side. The correlation between the epiphyseal tilt and alpha angle was statically significant (*p* = 0.017) with a medium effect size of 0.28; (4) Conclusions: The postoperative posterior epiphyseal tilt was predictive factor to determine the alpha angle. However, the cut-off value of the slip angle was 16.8° for a later occurrence of a FAI type cam indicating a small range of acceptable deviations from the anatomical position for SCFE reconstruction.

## 1. Introduction

The slipped capital femoral epiphysis (SCFE) is a hip disorder in the immature skeleton with an incidence between 0.33–24.85/100,000 [1]. In most cases the capital femoral epiphysis slides from the metaphysis in a medial-posterior direction (varus), but lateral-posterior displacements (valgus) are also described [2]. While an atypical type is caused by a known trigger including endocrine disorders, renal osteodystrophy or radiation treatment, the majority is idiopathic and only associated with general risk factors, especially obesity [3,4]. The affected children are on average between 11.7–13.0 years of age in dependence of the gender, causing females to usually sustain earlier a SCFE [5]. The disease occurs bilaterally in up to 80.0%, therefore the unaffected side can be treated in a prophylactic attempt [6]. The surgical procedures for the SCFE range from a closed reduction and internal fixation with wires or screws to open treatment options with osteotomies [7]. Independent of the chosen procedure, the overall aim is to restore and stabilize the anatomical position of the capital femoral epiphysis as close to natural as possible to achieve a physiological femoral neck junction and reduce the risk for osteonecrosis of the femoral head. A remaining incongruence and a remodeling process after the slip and the following treatment may cause a femoro-acetabular impingement (FAI) type cam in the early adolescence. The FAI was firstly described by Ganz et al. and represents a bony conflict between the acetabular rim and the femoral head-neck junction [8]. Generally, three types were differentiated. The FAI type pincer is defined as a local or general overcoverage of the femoral head due to prominence of the acetabular rim (for example in case of a retroversion, coxa profunda). The femoral deformity, the most common type, is described as FAI type cam and represents a bone thickening at the femoral head-neck junction leading to a mechanical conflict. The third type is a mixed type combining an acetabular and femoral deformity [9]. On the one hand the severity of the displacement (moderate, mild, severe) correlates with the rate of a FAI development, on the other hand a post-slip remodeling might influence the remaining femoral neck deformity [10,11]. According to this, the rate of the occurrence of a post-slip FAI was less common in patients younger than 11 years and might be explained by an extended time period of remodeling potential [12]. The FAI type cam is defined with an elevated alpha angle >55.0° (physiological range < 55.0°), which represents an angle between the femoral neck axis and a line connecting the center of the femoral head and the point of the bony head contour with a greater distance to the center than the radius [13]. Overall, the rate of FAI associated hip osteoarthritis was described with up to 91.0% in SCFE patients, compared to 43.0% in non-SCFE patients [10]. Fraitzl et al. reviewed 16 mild SCFE patients and observed a rate of elevated values of the alpha angle in all cases ranging from 55 to 99° [14]. The local bony thickening at the femoral neck leads to groin pain by damaging the acetabular labrum and the cartilage [15,16,17]. If left untreated, a FAI type cam might be responsible for an early onset osteoarthritis of the hip [18]. With a reshaping procedure of the femoral neck junction in an arthroscopic, arthroscopically-assisted or open setting, the progression of the joint degeneration may be prolongated and the total hip arthroplasty (THA)—free life time enhanced [19]. Hence, it is important to know which patients with a SCFE in their history are predestinated to develop a FAI type cam. With this information, the care of high-risk patients can be scheduled regularly over years to detect a symptomatic bony bump in an early stage. Further, in previous literature also an association between a SCFE and the development of a dysplastic socket shape and otherwise severe slips and a coxa profunda was observed [20,21].

Thus, the first aim was to detect the prevalence of pathological hip anatomies after SCFE (1). Another aim of this study was to analyze predictive factors in patients with a SCFE that may cause development of a FAI type cam over time (2).

## 2. Materials and Methods

This retrospective study was approved by the local ethic committee (No. 8779, Hanover Medical School, 28 November 2019). The inclusion criterium was a surgical treatment of a SCFE between 1 January 2005–31 December 2017 in our clinic (Department of Orthopaedic Surgery at Diakovere Annastift, Hannover Medical School) and included 113 cases in 111 patients. Exclusion criteria were missing radiological follow-up examination (e.g., due to outside follow-up examinations) or disapproval for participation. According to this, 18 cases (15.9%) resp. 15 patients (3 patients with a bilateral SCFE, 12 patients with a unilateral SCFE) were excluded from the study due to missing follow-up radiographs (at least two years) for the analysis of FAI type cam predicting factors. All patients were treated either with wires or with screws. The diagnosis was made with a radiological examination in two planes, pelvic anterior-posterior (a.p., standardized in supine position) and lateral views on both sides. All patients received postoperative physiotherapy training, including unloading or partial weight bearing for 6 weeks, depending on the patient’s age, weight and compliance. 

**Figure 1:**
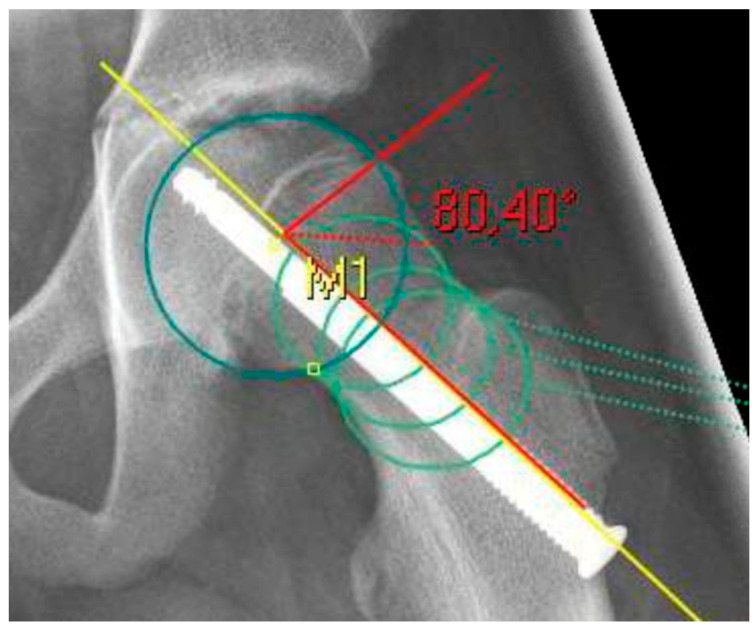
X-ray after screw fixation of a left sided SCFE as an example for the measurement of the alpha angle. The femoral neck axis (yellow line) was determined with three circles (light green) according to Bouma [29].

**Figure 2:**
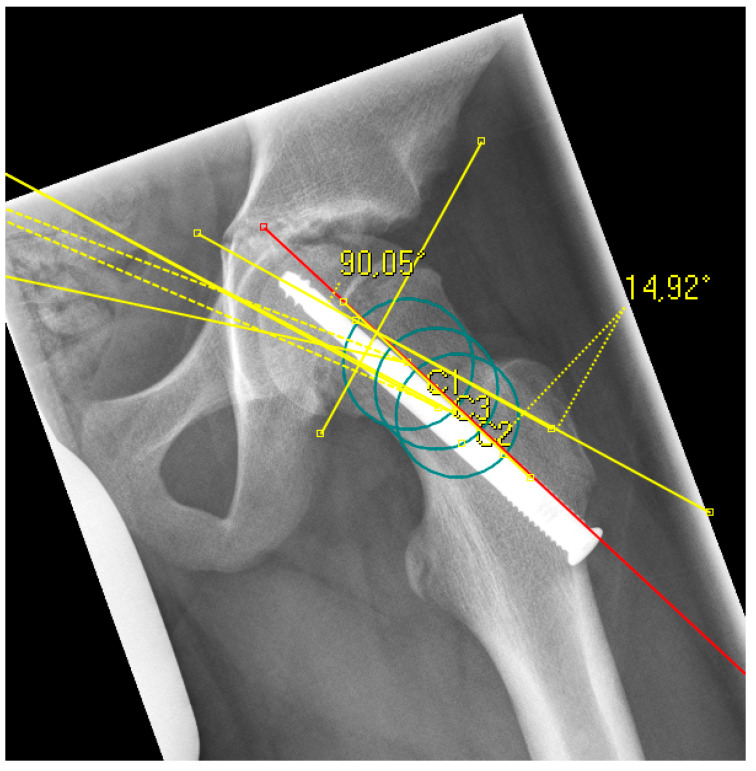
Example of a radiograph with the measurement of the epiphyseal tilt based on the anatomical femoral neck axis (red line), which was determined with three circles (green) according to Bouma [29].

The radiological analysis directly after surgical fixation included the lateral center edge angle according to the sourcil method (LCEA) [22] and the acetabular angle (inclination) to evaluate the femoral coverage [23], especially regarding the presence of a hip dysplasia or coxa profunda, as well as the absolute slip angle according to Southwick [24,25]. The grading of the Southwick angle values is typically: mild (<30°), moderate (30–50°) and severe (>50°). Because the alpha angle is referenced to the femoral neck axis, this reference was the same for the posterior epiphyseal tilt to predict a FAI type cam over the time [26]. In contrast to the Southwick angle, which is determined as an angle between the slipped capital epiphysis and the femoral shaft axis, the posterior epiphyseal tilt was referenced also to the femoral neck axis like the alpha angle [27]. In addition, parameters for dysplasia resp. retroversion were evaluated, including the crossover sign (the anterior and posterior acetabular rims crossing on the pelvic a.p.) and the prominence of the ischial spine (PRISS; the ischial spine is projected in the lesser pelvic on the pelvic a.p.) [28]. In presence of a crossover sign and a positive PRISS a retroversion was assumed, whereas a dysplasia was defined with a LCEA <20 and an inclination >10°. All the described values were assessed again at the last time of the radiological follow-up, additionally the alpha angle [13]. In presence of a SCFE, the center of the femoral head is not always aligned to the axis of the femoral neck for measurement of the alpha angle and the posterior epiphyseal tilt. To avoid inaccuracy, the anatomical method according to Bouma et al. was used [29]. For this, the femoral neck axis was defined with three circles along the femoral neck, whichever fitted the bony neck best (Figure 1 and Figure 2). 

The statistical analysis was performed with SPSS (IBM Corp., Armonk, NY, USA). After the descriptive analysis, an unpaired t-test was used for comparisons between the affected and the unaffected hip. To analyze the predictive impact of the postoperative slip angle and the posterior epiphyseal tilt on the alpha angle at the last time of follow-up, a linear regression was performed. The (Pearson) r describes the correlation between both values, which was categorized according to Cohen: 0–0.3 small; 0.3–0.8 medium; >0.8 strong correlation [30]. 

## 3. Results

From the 95 remaining cases, 91 children (97.8%; 41 right side/ 50 left side) sustain an unilateral and 2 (2.2%) showed bilateral SCFE at time of diagnosis. The total group included 46 boys (49.5%) and 47 girls (50.5%) with a mean age of 12.8 years (±2.2; 8.9–16.9) and a mean body mass index (BMI) 26.5 kg/m^2^ (±4.2; 20.4–33.3). 

The direct postoperative anatomy of the hip socket on the affected side was 29.6° (±6.8; 12.1–47.8) for the LCEA and 8.7° (±4.0; 1.2–20.0) for the acetabular angle. 37 patients (38.9%) presented dysplastic values for at least the LCEA, the acetabular angle or both. Further, radiological signs of a hip retroversion were in 21 cases (22.1%) a positive crossover and in 16 cases (16.8%) a present PRISS. On the unaffected side, 18 patients (18.9%) showed a hip dysplasia with an overall LCEA of 30.0° (± 6.4; 17.0–46.8) and a mean acetabular angle of 7.4 (±3.8; 0.5–22.4). The mean radiological follow-up of the 95 cases was 4.3 years (±1.9; 2.0–11.2), resulting in a mean age of approximately 17.0 years (±2.6; 11.1–26.5). Although the hip socket was not treated, the acetabular anatomy was measured at the last time of the radiological follow-up to observe the further possible changes of the coverage. According to that, 33 patients (34.7%) had at least one dysplastic socket angle on the affected side with an overall LCEA at the last examination of 27.6° (±7.9; 8.3–44.0) and an acetabular angle of 8.0° (±4.4; 0.1–20.3). From these patients, 21 (63.6%) showed already direct postoperatively dysplastic values. In spite of the small reduction of the acetabular values, the presence of a crossover sign increased to 53 cases (55.8%) and to 44 cases (46.3%) with a PRISS. 15 patients (15.8%) showed a hip dysplasia on the unaffected side at the last time of follow-up, two less than directly after surgery. The socket anatomy was on average with a LCEA of 30.7° (±8.7; 13.2–49.9) and an acetabular angle of 6.5° (±4.0; 0.4–19.7). The mean Southwick angle after surgery was 32.8° (±16.0; 2.3–76.1): 43 (45.3%) cases in a mild (0–30°) and 36 (37.9%) in a moderate (30–50°) and 16 (16.8%) in a severe (>50°) displaced position, whereas posterior epiphyseal tilt was 18.0° (±12.0; 1.0–49.6). At the last time of the radiological follow-up, the alpha angle was in 46 cases (48.4%) >60°, representing a FAI type cam. In the 93 cases with a unilateral SCFE, the alpha angle of the unaffected side was about 14.4° lower (50.0 ± 10.1 vs. 64.4 ± 17.7°) compared to the diseased hip (*p* = 0.001). 

**Figure 3:**
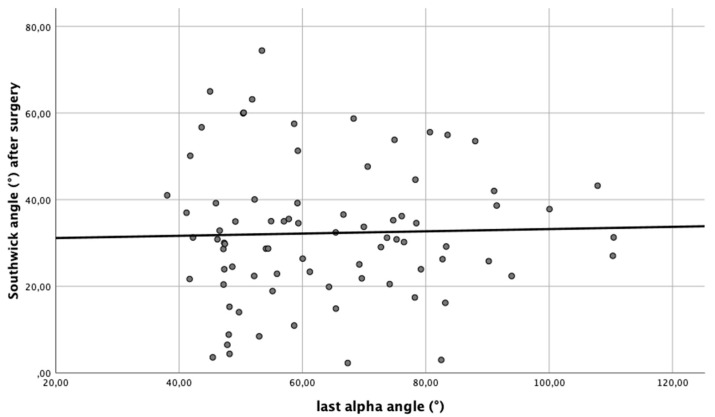
The linear regression between the postoperative absolute Southwick angle and the latest alpha angle showed no significant correlation.

The correlation between the direct postoperative Southwick angle and the alpha angle at the last time of follow-up was low (*r* = 0.03; Figure 3). Due to the missing correlation, no further observations or statistics were performed. 

**Figure 4:**
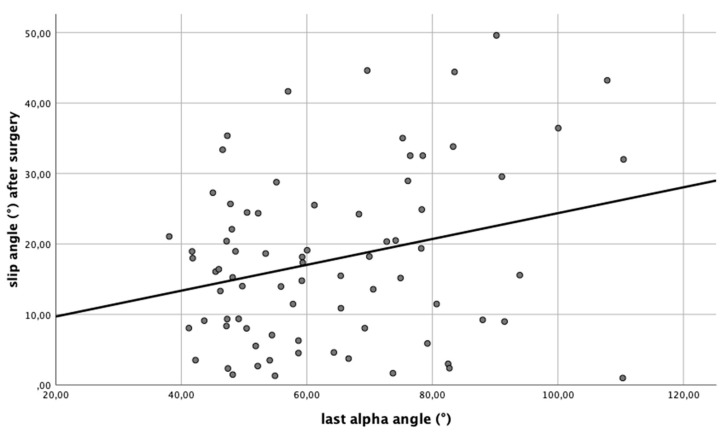
Linear regression y = 6.0 + 0.18x between the epiphyseal tilt (slip angle) immediately after surgery and the alpha angle at the last time of radiological follow-up. The cut-off value for the postoperative epiphyseal tilt, which was associated with a later cam-deformity occurrence (x = 60), was 16.8°.

In contrast to that, the regression between the posterior epiphyseal tilt and the alpha angle of the last radiograph indicated that from a posterior epiphyseal tilt above 16.8°, the estimated alpha angle over time was greater than 60°. The correlation was statistically significant (*p* = 0.017). Per 1° of the postoperative posterior epiphyseal tilt, the alpha angle increased by 6.18° in the last radiological examination (Figure 4). With a *r* = 0.27, a small correlation according to Cohen is present. 

At the time of surgery, 8 patients (8.4%) showed a hip dysplasia on the affected side determined by a LCEA below 20°, whereas 37 patients (38.9%) had an acetabular index above 10°, including 6 patients (6.3%) with both characteristics. Compared to the unaffected side, the hip joint with the SCFE condition showed a significantly decreased coverage for both parameters (Table 1). However, a statistically significant correlation between the severity of the SCFE and the presence of dysplastic socket angles was not observed (*p* > 0.05). 

## 4. Discussion

During childhood, the SCFE is one of the most prevalent hip disorders, and due to the occurrence in the immature skeleton, the complications in the joint function might extend to further life stages. In spite of an adequate reconstruction, an incongruence of the femoral head neck junction remained in many patients [7]. Experience of pain and limitations in range of movement (ROM) caused by joint impingement, might provide an indication in performing preserving arthroscopies or open procedures. However, it is estimated that the rate of THA after SCFE is about 22.0–45.0% in middle age [31,32]. Thus, the current study analyzed the rate of hip deformities following a SCFE and predictive factors detecting patients with a high risk for the development of a FAI over time. Further, regarding the coverage the evidence in the international literature is limited and not consistent as ranging from associations with dysplastic and deep sockets [20,21]. 

First of all, the cohort include common findings regarding the age and BMI. According to this, the mean age was 12.8 years and in range with other population-based results (11.7–13.0) [5]. Further, the mean BMI was 26.5 kg/m^2^ and represents under recognition of the mean age a percentile value > 95.0%, which also was observed in other studies [4,33]. However, the focus of this retrospective study was the anatomy of the hip socket and the femoral head-neck junction over time after SCFE to determine the frequency of a FAI type cam and a trend to a more retroverted and dysplastic socket shape compared to the unaffected hip joint. The radiographs of the last examination presented an elevated alpha angle >60° in 48.4% of the cases and compared to the unaffected side, the alpha angle was about 14.4° higher at the joint side sustaining a SCFE (*p* < 0.001). In addition, the patients with an increased alpha angle (>60°) at the last follow-up showed a significant higher perioperative posterior epiphyseal tilt (15.1 ± 9.8 vs. 20.7 ± 13.8; (*p* < 0.01)). The determined postoperative cut-off value for the reconstructed epiphyseal tilt resulting in a radiological appearance of a FAI type cam (alpha angle > 60°) was 16.8°. Although the correlation between both values was statistically significant (*p* < 0.05), the predictable amount was limited with r = 0.27. However, the postoperative Southwick angle showed no correlation to the latest alpha angle and as such did not present a predictive factor for the occurrence of a FAI type cam over time. Similar results were found by Dodds et al. [34]. The authors analyzed predictive factors for a cam deformity after SCFE in 46 patients. The perioperative slip angle at r = 0.17 showed a poor correlation to the follow-up alpha angle, so that the authors recommended to monitor all SCFE patients into adulthood for an impingement [34]. Another study also demonstrated a non-significant trend that preoperative slip angles >35° were associated with a later onset of an impingement of the hip [35]. Furthermore, the a.p. alpha angle was found as predictive factor for a poor clinical (HHS < 85 points) as well as radiological follow-up result by Terjesen et al. [36]. In analogy with that, a dysplastic socket anatomy was found in 8.4% with a LCEA <20° and in 38.9% with an acetabular angle >10°. Furthermore, the affected hip had a decreased coverage (LCEA/acetabular angle: 27.6 ± 7.9/8.0 ± 4.4) compared to the healthy side (LCEA/acetabular angle: 30.7 ± 8.7/6.5 ± 4.0) (*p* < 0.001). In spite of statistical significance, the clinical relevance might be low, especially because both mean values represent sufficient coverage. The differences in the determined rates of a dysplastic hip anatomy in dependence of the chosen angle, LCEA or acetabular angle, might be explained by the way of the measurement. For the determination of the LCEA, the center of the femoral head is required, but the displacement in case of SCFE may lead to a deformed and noncircular shaped femoral head, resulting in an inaccuracy for the determination of the femoral head center while LCEA measurement. Another explanation might be the chosen cut-off values. Defining a dysplastic anatomy with LCEA <25.0° and an acetabular angle >12.0° might has adjusted the values. However, Maranho et al. also observed in their longitudinal study of 108 patients a tendency to a decreased femoral head coverage of SCFE affected hips [20]. It is supposed, that the epiphyseal displacement might influence the physiological growth of the acetabular socket. According to this, the remodeling after the slip might lead to a relative overgrowth of the femoral head leading to dysplastic conditions or in presence of a FAI the bony might impair the ossification of the acetabular rim [37,38]. Otherwise, some studies observed overcovered anatomies and deep sockets in patients with SCFE [39,40]. In congruence with that, in the time period directly after surgery and to the last radiological follow-up an increase of signs for a retroverted acetabulum was observed. According to that, the among of positive crossover signs raised from 22.1% to 55.8% resp. for a PRISS from 16.8% to 46.3%. In spite of a standardized supine positioning in our institute, the pelvic tilt was not evaluated with the sacrococcygeal-symphysis distance, which might have influenced these results. However, the association between hip pathologies and the presence of an acetabular retroversion was observed. In comparison to the physiological retroversion prevalence of approximately 6%, Bauer et al. found at least one sign (crossover or PRISS) in 79–82% bilateral resp. unilateral involved SCFE hips [41,42]. These observations were not constantly proofed by other studies [43,44]. The presented results with follow-up examinations showed a hint for a development to an acetabular retroversion in comparison to the direct postoperative results and to the uninvolved side in unilateral cases. However, the association between an acetabular retroversion and a SCFE needs further studies to be proofed. 

The study has some limitations. The first aspect was the limited radiological follow-up about 4.3 years (±1.9; 2.0–11.2). As a result, deviations of the physiological hip anatomy developing later in the patient’s life might not be detected in this study (information bias). This could explain the difference in the rates of detected FAI type cam over the time, 48.4% in the current work, compared to 91.0% in candidates for THA [10]. However, the mean age at time of the last radiological follow-up was approximately 17.0 years meaning that the majority of the cohort were followed-up after skeleton maturity. In addition, for young patients with a constant level of their known symptoms after SCFE, frequent radiological follow-up should be carefully discussed regarding radioprotection. Thus, there might be a lack of radiographs to show increasing disabilities between the ages of 20–35 years. Lack of radiation might also explain the usage of conventional radiographs in the current study, although with SCFE representing a three-dimensional slip, a more precise analysis could be performed in CT. However, the indication for a CT in young patients should stay an exception and not be routinely performed. This work analyzed the radiological presence of a bone thickening at the head-neck junction without information about the clinical symptoms or functional limitations of the observed patients. Another limitation was the observational study design resulting in associated biases. According to this the current study has not a comparative character due to a missing patient’s group with physiological hip conditions (selection bias) [45,46]. However, in the current study the unaffected side of patients sustained from a unilateral SCFE was analyzed and worked as comparative dataset. Another aspect of observational studies is the confounding [45,46]. While the association between a SCFE and a development of a FAI type cam over time was proofed in this and previously in other studies, the associated socket morphology is currently discussed in literature and included hints for deep as well as insufficient, dysplastic covering hips [20,39,40]. These inconsistent observations might be caused by a confounding factor. 

## 5. Conclusions

The postoperative posterior epiphyseal tilt represented a limited predictive factor to determine the alpha angle over time in patients sustaining a SCFE. However, the cut-off value of the epiphyseal tilt for a later occurrence of a FAI type cam was about 16.8°, indicating a small range of acceptable deviations from the anatomical position for SCFE reconstruction. Because there are no clear predictive factors for the occurrence of an FAI, all patients sustained from SCFE should be followed-up at least once after skeleton maturity.

## Figures and Tables

**Table 1:** 41 patients (43.2%) from the total group (*n* = 93 with unilateral SCFE) had a dysplastic socket anatomy on the affected side with at least one of the following criteria: LCEA < 20.0°, acetabular angle > 10.0°. At the last time of follow-up, the affected side had a decreased coverage compared to the unaffected hip. However, the mean values of both sides were in a physiological range and the clinical relevance might be limited with differences between 1.5° (acetabular angle)–3.1° (LCEA).

	Affected Side	Unaffected Side	*p*
LCEA	27.6 ± 7.9	30.7 ± 8.7	0.001
Acetabular angle	8.0 ± 4.4	6.5 ± 4.0	0.001

## Data Availability

The data presented in this study are available on request from the corresponding author. The data are not publicly available due to privacy protection.
